# Common Vetch: A Drought Tolerant, High Protein Neglected Leguminous Crop With Potential as a Sustainable Food Source

**DOI:** 10.3389/fpls.2020.00818

**Published:** 2020-06-19

**Authors:** Vy Nguyen, Samuel Riley, Stuart Nagel, Ian Fisk, Iain R. Searle

**Affiliations:** ^1^School of Biological Sciences, School of Agriculture, Food and Wine, The University of Adelaide, Adelaide, SA, Australia; ^2^Shanghai Jiao Tong University Joint International Centre for Agriculture and Health, The University of Adelaide, Adelaide, SA, Australia; ^3^South Australian Research and Development Institute, Adelaide, SA, Australia; ^4^Division of Food Science, Nutrition and Dietetics, School of Biosciences, University of Nottingham, Nottingham, United Kingdom

**Keywords:** legume, common vetch, *Vicia sativa*, vetch toxin, γ-glutamyl-β-cyano-alanine, plant-based protein, sustainable

## Abstract

Global demand for protein is predicted to increase by 50% by 2050. To meet the increasing demand whilst ensuring sustainability, protein sources that generate low-greenhouse gas emissions are required, and protein-rich legume seeds have the potential to make a significant contribution. Legumes like common vetch (*Vicia sativa*) that grow in marginal cropping zones and are drought tolerant and resilient to changeable annual weather patterns, will be in high demand as the climate changes. In common vetch, the inability to eliminate the γ-glutamyl-β-cyano-alanine (GBCA) toxin present in the seed has hindered its utility as a human and animal food for many decades, leaving this highly resilient species an “orphan” legume. However, the availability of the vetch genome and transcriptome data together with the application of CRISPR-Cas genome editing technologies lay the foundations to eliminate the GBCA toxin constraint. In the near future, we anticipate that a zero-toxin vetch variety will become a significant contributor to global protein demand.

## Introduction

Global demand for protein is predicted to increase by a staggering 50% by 2050 (Westhoek et al., 2011; Henchion et al., 2017). With an increasing global population and increasing demand for animal-derived protein, the sustainability of agriculture systems has been brought into question. Over the last two centuries, the expanding livestock industry has led to significant deforestation, and overgrazing of natural grassland environments such that it has caused decreased terrestrial biodiversity and increased greenhouse gas emissions and contributed to climate change and global warming (Henchion et al., 2017). In order to meet the increasing protein demand and protect our environment, more sustainable protein food sources are required. Cheap plant-based protein, such as legume seeds, represent an environmentally sustainable option that is well suited for developing countries with rapidly growing populations (Asgar et al., 2010). Moreover, to cope with increasingly unpredictable climate change and expansion of marginal cropping areas, breeding strategies for more drought tolerant and resilient crops will be vital (Sivakumar et al., 2005; Lobell and Gourdji, 2012). A legume that could be exploited for this scenario is the common vetch (*V. sativa*). Common vetch is able to grow in marginal cropping zones whilst being resilient to variable annual weather patterns mainly through superior drought tolerance (White et al., 2005). One study demonstrated that vetch could withstand water deficit for up to 24 days and show full restoration of biotic function once regular watering had resumed (Tenopala et al., 2012). Drought and heat tolerant crops are increasingly desirable in the face of rising global temperatures and increasingly prolonged periods of drought brought on by climate change (Mba et al., 2018).

For many decades, the inability to remove the γ-glutamyl-β-cyano-alanine (GBCA) seed toxin has hindered common vetch’s use in agriculture (Pfeffer and Ressler, 1967; Roy et al., 1996) leaving this resilient plant as an “orphan” legume. We envisage that the development of a zero-toxin vetch variety would facilitate its use for animal feed, specifically chickens and pigs, and human consumption (Ressler et al., 1997; Collins et al., 2002). We estimate the production costs of common vetch are approximately 50% less than competing legumes such as lentils and predict that zero-toxin varieties would rapidly surpass lentil in pig and poultry production. Zero-toxin common vetch will immediately generate new domestic markets, such as feed for the poultry industry, but it will also open new export markets for Australia and other countries thereby increasing export revenue and increasing farm profitability and indirectly increase investment for their local communities.

## Common Vetch: A Versatile Pasture Crop That Provides Multiple Benefits for the Farm

**FIGURE 1:**
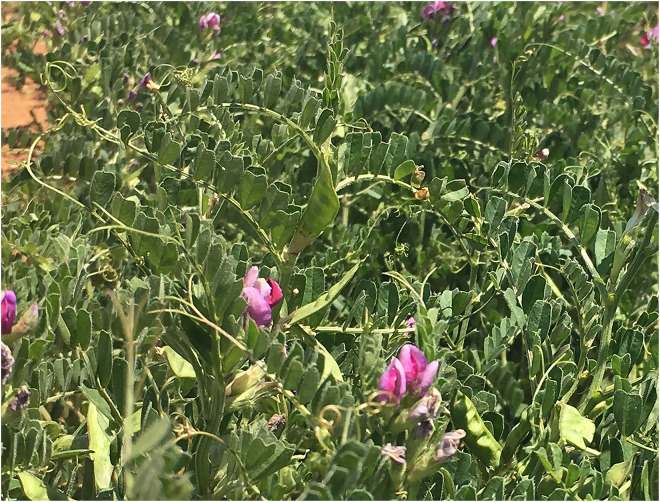
Field grown *Vicia sativa* growing at the National Vetch Breeding Program in South Australia.

Common vetch (*V. sativa*) which is shown in Figure 1 belongs to the *Fabaceae* (legume) family, within the genus *Vicia*. This genus contains about 140 species including woolly-pod vetch (*V. villosa*) and faba bean (*V. faba*). Other *Fabaceae* genera also contain so-called vetches; of which two examples are *Astragalus* (containing the milkvetches) and *Lathyrus* (containing *L. ochrus*, the cyprus-vetch). Nowadays, common vetch is commonly found both in natural and agricultural settings across Europe, Asia, North America, some parts of South America, Africa, the Mediterranean, and Australia (Navrátilová et al., 2003; Ford et al., 2008).

Like other legumes, common vetch forms a symbiosis with nitrogen-fixing bacteria (*Rhizobia*) that fix atmospheric nitrogen into nitrogenous compounds available to the plant, hence reducing the need for application of expensive nitrogen fertilizer and subsequent rotation crops. Often, common vetch is used as a green manure which, when incorporated into the soil, provides valuable carbon, and nitrogen for rotation crops such as wheat and barley. Additional soil carbon often increases water-holding capacity and ability to bind nutrients including nitrate (Reeves, 1997; Bünemann et al., 2018). Furthermore, common vetch biomass can also be used for forage, fodder, pasture, silage, or hay and the seed may safely be used as a protein-rich feed component for ruminant animals (Enneking, 1995). Common vetch is well suited as a pasture species as it forms many adventitious shoots that are either buried or close to the soil surface thus giving it the ability to be resilient to heavy grazing (Rathjen, 1997).

**FIGURE 2:**
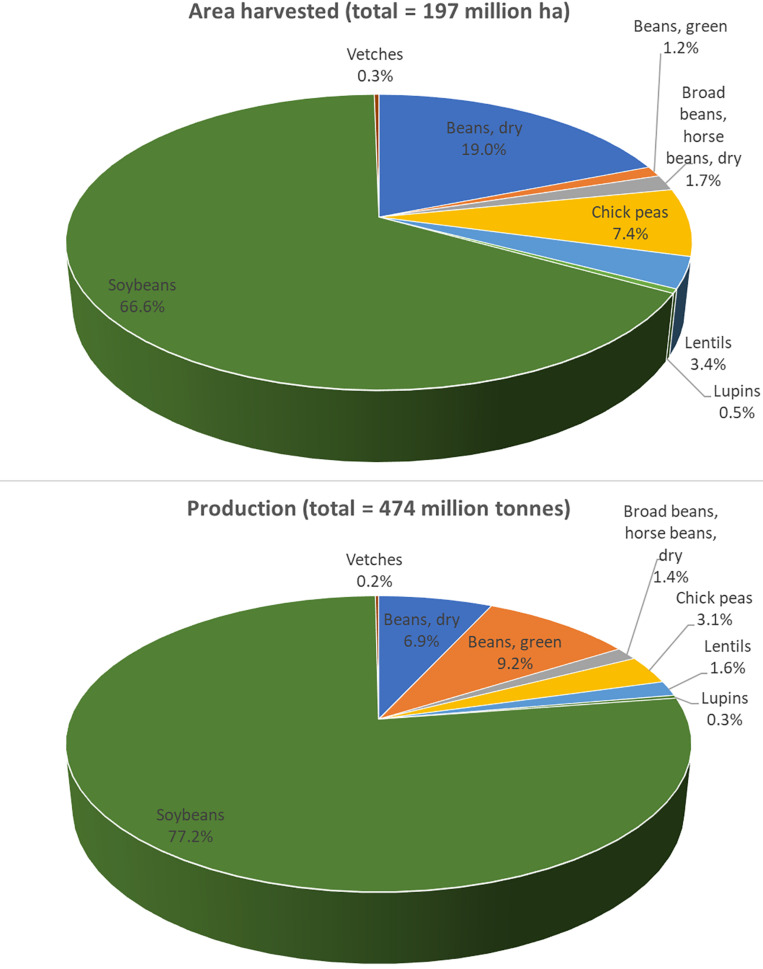
Common vetch production when compared to other legumes globally in 2017. In general, common vetch occupied about 0.3% of the area harvested and 0.2% of the production. For vetch, total area harvested in 2017 was 560,077 ha and total production was 920,536 tons. Data was published by the Food and Agriculture Organization (FAO; http://www.fao.org/).

Despite common vetch’s versatile uses, the production of vetch is still limited. Data collected by the Food and Agriculture Organization (FAO) in 2017 showed that, globally, the area harvested and the production of vetch were about 0.6 million ha and 0.9 million tons, respectively^1^. Based on FAO 2017 data for legumes, vetch occupied about 0.3% of land usage and accounted for only 0.2% of the production. This is 12 times less than the area harvested and 8 times less than the production of lentils (Figure 2). Common vetch’s limited production is mainly attributed to the anti-nutritional compounds existing in the seeds which will be further discussed in the next section.

## Common Vetch Seeds: Important Nutritional Attributes

Common vetch seed appeared in the diets of hunter-gatherers as early as 12,000–9,000 BP as evident in archaeobotanical analysis of samples from the Santa Maira cave in Alicante, Spain (Aura et al., 2005; Mikić, 2016). Today, common vetch is globally distributed and its spread is thought to have occurred through the inadvertent selection and trading of vetch seeds as a weedy contaminant with other legume seeds (Erskine et al., 1994). This has led to the suggestion that selection of common vetch amongst other palatable legumes, such as lentils, led to vegetative, and seed mimicry of vetch to lentils (Erskine et al., 1994). Although intact lentil and vetch seed can be differentiated after close examination of the seed shape and size, the split seed is harder to distinguished. Due to the similarity of split vetch and lentil seeds, there was a period of time when split vetch seeds of the variety “Blanchefleur” were oil coated and inappropriately substituted and sold as lentils. However, Tate and Enneking (1992) raised the so-called substitution issue of vetch toxicity and indicated that vetch seeds were unsuitable to be eaten by monogastric animals, including humans. Since then, vetch has been restricted to use in pastures as the seeds can be safely eaten by ruminants and some monogastric animals, such as chicken (less than 40% w/w), and pig (less than 20% w/w) in low amounts (Rathjen, 1997).

Common vetch exhibits high concentrations of crude protein (w/w) in the seed between 24 to 32% (Francis et al., 2000). This concentration is comparable to faba bean (*V. faba*) and lupin (*Lupinus angustifolius L.*) seed which have about 32% (Crépon et al., 2010; Caliskanturk et al., 2017) and 30% crude protein (Valentine and Bartsch, 1996), respectively. Common vetch seeds contain eighteen amino acids and the ratio of essential amino acids/non-essential amino acids is about 0.7 (Mao et al., 2015) which is significantly higher than the 0.38 recommended by WHO (Joint, 2007). Additionally, the principle essential amino acids are arginine and leucine at average concentrations of 2.4 and 2.1%, respectively. Glutamic and aspartic acids are the predominant non-essential amino acids within common vetch seed, averaging levels of 5.5 and 3.7%, respectively (Mao et al., 2015). Vetch seed also contains a much lower proportion of lipids (only 1.5–2.7%) compared with soybean seed (*Glycine max*) and this is primarily composed of unsaturated fatty acids (Mao et al., 2015).

Presently, common vetch is mainly used as animal feed for ruminants and it can be argued that higher value returns could be observed through the repurposing of common vetch as a human food crop. Obstructing the development of the higher value product is a range of anti-nutritional factors, the most significant of which are the dipeptide GBCA and the free amino acid β-cyano-L-alanine (BCA), which exist in relatively high concentrations in the seed at approximately 2.6 and 0.9%, respectively (Tate et al., 1999). Such compounds are toxic to monogastric species, such as chickens or pigs, but have no obvious effect upon ruminant species, including beef cattle (Valentine and Bartsch, 1996). The proportion (w/w) of common vetch seed within feeds that can be safely consumed without deleterious effects is 10% for piglets and 20% for adult pigs and for chickens about 40% (Harper and Arscott, 1962; Rathjen, 1997). In addition to the main toxin GBCA, other anti-nutritional compounds were also found in common vetch included vicianine, vicine, convicine, and tannins (Ritthausen and Kreusler, 1870; Rathjen, 1997).

Genetic variation and relatively high heritability of vicianine levels in common vetch accessions have allowed breeders to select and produce cultivars free of this toxin. One early developed cultivar with no vicianine was Blanchefluer (Delaere, 1996; Rathjen, 1997). Most modern common vetch cultivars are vicianine free. Vicine and convicine are well-studied in faba bean, and in humans cause the potentially fatal disease favism (Bottini, 1973). Vicine and convicine are hydrolysed by native β-glucosidases in the cotyledons of seeds to form divicine and isouramil and when consumed by humans can cause oxidation of glutathione in red blood cells. In individuals who cannot generate glutathione at normal rates due to a deficient glucose-6-phophatedehydrogenase activity this results in haemolysis and the disease favism (Arese et al., 1981). Unlike vicianine and vicine, wild accessions and breeding lines with very low GBCA levels have not been identified, and the GBCA toxin levels in current cultivars is still deemed to be too high for monogastric consumption. Chickens can only tolerate feed with less than 20% (w/w) common vetch (∼0.2% GBCA in the feed). Confounding toxic effects of other compounds with vicine and GBCA were proposed (Rathjen, 1997), however, the chemical basis of these compounds is currently unknown. Finally, anti-nutritional tannins present in vetch seed coats are often removed during the dehulling process and hence are considered less significant (Rathjen, 1997).

## Limitation of GBCA Detoxification Using Conventional Methods

Without the toxic compounds in the seeds, vetch would be highly nutritious and a valuable animal feed. Therefore, a number of methods have been investigated to remove the toxins, mainly focusing on GBCA. Post-harvest processing efforts to lower the GBCA toxin levels within the seed have previously involved simple soaking, continuous flow through soaking, and boiling methods (Rathjen, 1997). The seed soaking method alone was insufficient to lower GBCA levels as consumption of soaked seed during feeding trials by chickens reduced egg production, and daily food consumption and feed conversion ratios were also diminished (Farran et al., 1995). In contrast, the boiling method reduced the toxin levels in the seed such that the seed could be included in chicken feed at levels of up to 25% without negative effects on growth rates (Kaya et al., 2013). However, consumption of boiled vetch seed that had the broth periodically discarded during boiling resulted in 20% reduced growth rates in chickens when compared with conventional feed with similar protein amounts (Ressler et al., 1997). This boiling method combined with periodically discarded water had 45% decreased seed mass as water soluble vitamins, like vitamin B, water soluble proteins and carbohydrates were leached during processing (Ressler et al., 1997), and this correlated with the reduced chick growth rate. Autoclaving the seed as a processing method has also been investigated and assessed in feeding trials of laying hens. In the laying hen trial, overall growth rate was found not significantly different between animals fed with autoclaved or raw vetch seed suggesting that the non-heat labile toxins like GBCA were still bioactive (Farran et al., 1995). The lack of success of these seed processing methods in improving animal growth or health has strongly indicated the need for genetic approaches to detoxify the common vetch seeds.

## Unsuccessful Searches for Zero-Toxin Vetch Accessions

Using conventional breeding methods and more recently the use of molecular marker-assisted breeding for genomic selection, plant breeders have prioritized the search for common vetch varieties that have biotic and abiotic stress resistant traits as well as selecting for increasing yield and seed nutritional quality (Francis et al., 2000). However, no concerted effort has been made to select for low or zero GBCA toxin levels in Australian or overseas breeding programs. This has resulted in varieties that are only used by farmers for pasture, green or brown silage, or ruminant feed (Francis et al., 2000; Dong et al., 2019; Huang et al., 2019; Mikić et al., 2019). This is mainly due to very limited natural variation in GBCA toxin levels amongst common vetch accessions (Rathjen, 1997). Rathjen (1997) screened over 1,700 *V. sativa* accessions and failed to identify a single accession with no GBCA toxin. Later, screening of a total of 3,000 accessions identified only one line, IR28, with a low (0.3–0.4%) GBCA level but no zero-toxin line has yet been identified (Ford et al., 2008). Backcrossing IR28 to Jericho white, a spontaneous white flowered mutant of the French commercial variety Languedoc, over seven generations produced a near homozygous line named Lov 9 (Tate and Searle, unpublished). However, the GBCA levels in the Lov 9 seed from plants grown in shade houses or field conditions ranged from 0.4–1.2%, respectively (Tate and Searle, unpublished). These GBCA levels in Lov 9 seed were deemed too high for commercial release of the variety as a low toxin variety. Another strategy to develop a zero GBCA toxin common vetch variety was interspecies crosses of zero toxin species *V. villosa* and *V. pannonica* to common vetch but these resulted in embryo abortion and no viable hybrids were recovered (Searle, unpublished). Considering the limited success to date, other pathways to produce a zero-toxin common vetch variety are required.

## Application of Biotechnology to Produce Zero-Toxin Vetch

Applications of biotechnology have promised to accelerate crop improvement (Moose and Mumm, 2008). The emergence of genomics, transcriptomics, metabolomics, and proteomics data has led to the establishment of publicly available databases for most major crops. For example, the LIS – Legume Information System (Dash et al., 2015), and eFP browser (Patel et al., 2012; Hawkins et al., 2017) now contain data for legumes. By combining the information available in these databases with new bioinformatic tools, we now have the ability to dissect complex traits to determine the underlying gene architecture in a more comprehensive way. In 2018, the 1.8 Gb common vetch genome and seed transcriptome sequencing projects were initiated at the University of Adelaide, Australia, opening the opportunity to determine the genetic basis of the vetch toxin accumulation. Using this transcriptome data, we could identify the genes involved in toxin production and in the future we could investigate their functions by overexpressing or mutating candidate genes. Moreover, we envisage that application of CRISPR-Cas (clustered regularly interspaced short palindromic repeats – Cas protein) genome editing to modify agronomically important traits in crops such as wheat, barley, rice, and tomato (Liu et al., 2017) will soon be applied to more challenging species including the common vetch. Using CRISPR-Cas genome editing, the nutritional profiles of many crops have been recently demonstrated. For example, in tomato, knocking down genes in the carotenoid metabolic pathway led to a 5-fold increase in lycopene (Li et al., 2018), and in rice, generating mutations in the starch branching enzyme (Berrens et al., 2017) genes increased amylose content by up to 25% and resistant starch to 9.8% (Sun et al., 2017). One of the most significant impacts of CRISPR-Cas genome editing is the potential improvement of a key trait in a commercially released cultivar within 6 months. In contrast conventional breeding of the trait may take 5–7 years to release the new cultivar. Importantly, it only takes one generation to obtain an edited plant using genome editing.

A major challenge in non-model plant systems like common vetch, is the delivery of transgenes such as CRISPR-Cas ribonuclear complex into plant cells and subsequent plant regeneration. Unlike crops such as rice and barley where the transformation and plant regeneration systems are standardized (Sahoo et al., 2011; Harwood, 2014), efficient transformation, and plant regeneration systems are lacking for common vetch (Ford et al., 2008). Unfortunately, common vetch’s GBCA toxin level have lowered the priority for research and development of these necessary biotechnological tools – for example developing a transformation system. Further investment in common vetch is required to develop transformation and plant regeneration systems to facilitate the application of genome editing for trait improvement.

## Future Work and Expectations

The environmental benefits, the versatile growth habit, and the rich nutritional profile of common vetch make the legume an appealing crop to meet future protein food requirements for humans and animals while sustainably contributing to our agricultural system. However, the failure of conventional breeding to develop a zero-toxin common vetch variety requires a new strategy to be employed. The recent availability of new vetch genomic resources and tools for genome editing increase the likelihood of solving the vetch GBCA toxicity problem. To make this plausible, we suggest the following steps are important for vetch.

To solve the vetch GBCA toxicity, we suggest the following experiments should be prioritized:

1. Sequencing, assembling and annotating the genome.
2. Comparing gene expression by RNA-seq and undertake biochemical pathway analysis of differentially abundant genes across vetch cultivars and *Vicia* species that do not contain GBCA.
3. Identifying candidate GBCA detoxifying genes and undertake functional gene analysis.
4. Establishing an efficient transformation and plant regeneration system.
5. Applying CRISPR-Cas9 or gene expression cassettes of candidate gene(s) to manipulate gene expression levels.
6. Screening of zero-toxin vetch lines and testing in animal assays.

Although the first three objectives above can be readily achieved, the last three could take many years to be accomplished due to the absence of a demonstrably routine *in vitro* transformation and regeneration system for vetch. Therefore, expertise from the plant tissue culture field will be invaluable to achieving objective four and ultimately the final goal.

## Data Availability Statement

The datasets generated for this study are available on request to the corresponding author.

## Author Contributions

VN and IS initially conceived the manuscript. All authors contributed to the writing and editing of the manuscript.

## Conflict of Interest

The authors declare that the research was conducted in the absence of any commercial or financial relationships that could be construed as a potential conflict of interest.

## Footnotes

1 http://www.fao.org/faostat/en/#data
